# Co-Speech Hand Gestures Are Used to Predict Upcoming Meaning

**DOI:** 10.1177/09567976251331041

**Published:** 2025-04-22

**Authors:** Marlijn ter Bekke, Linda Drijvers, Judith Holler

**Affiliations:** 1 Donders Institute for Brain, Cognition, and Behaviour, Radboud University; 2 Max Planck Institute for Psycholinguistics, Nijmegen, the Netherlands

**Keywords:** multimodal communication, language, prediction, co-speech gesture, EEG

## Abstract

In face-to-face conversation, people use speech and gesture to convey meaning. Seeing gestures alongside speech facilitates comprehenders’ language processing, but crucially, the mechanisms underlying this facilitation remain unclear. We investigated whether comprehenders use the semantic information in gestures, typically preceding related speech, to predict upcoming meaning. Dutch adults listened to questions asked by a virtual avatar. Questions were accompanied by an iconic gesture (e.g., typing) or meaningless control movement (e.g., arm scratch) followed by a short pause and target word (e.g., “type”). A Cloze experiment showed that gestures improved explicit predictions of upcoming target words. Moreover, an EEG experiment showed that gestures reduced alpha and beta power during the pause, indicating anticipation, and reduced N400 amplitudes, demonstrating facilitated semantic processing. Thus, comprehenders use iconic gestures to predict upcoming meaning. Theories of linguistic prediction should incorporate communicative bodily signals as predictive cues to capture how language is processed in face-to-face interaction.

In face-to-face conversation, people use speech and gesture together to convey meaning. For example, people produce hand movements that depict concrete concepts, such as wiggling the fingers to depict typing (i.e., iconic gestures). Comprehenders integrate the meaning from speech and gesture, and, importantly, seeing gestures alongside speech facilitates their language processing compared with only hearing speech. Behavioral evidence for such facilitation shows that seeing hand gestures is associated with faster responses, both in natural conversations (Holler et al., 2018; Kendrick et al., 2023; ter Bekke et al., 2024b) and controlled experiments (e.g., Kelly et al., 2010; Krason et al., 2022; ter Bekke et al., 2024a). Moreover, neuroscientific studies have shown that seeing congruent iconic gestures alongside speech facilitates processing of the speech, as indexed by less negative N400s (e.g., Hintz et al., 2023; Osorio et al., 2024; Wu & Coulson, 2010; Zhang et al., 2021). Crucially, the mechanisms underlying this gestural facilitation remain unclear. We investigated the hypothesis that comprehenders may use the semantic information conveyed by gestures, which typically precedes related speech, to predict upcoming meaning (Holler & Levinson, 2019; Skipper, 2014; ter Bekke et al., 2024b).

In line with this hypothesis, a large body of evidence shows that comprehenders use preceding context to predict upcoming input while processing speech (e.g., Huettig, 2015; Kuperberg & Jaeger, 2016; Pickering & Gambi, 2018). However, previous work has almost always focused on preceding speech context, and very little is known about whether visual bodily signals can be used for prediction. Gestures could be part of this preceding context used for prediction (Holler & Levinson, 2019; Skipper, 2014) because in face-to-face conversation—the natural environment of language use—gestures typically already depict semantic information before their corresponding speech starts (e.g., Schegloff, 1984; ter Bekke et al., 2024b). More specifically, we define prediction as context changing “the state of the language processing system before new input becomes available, thereby facilitating processing of this new input” (Kuperberg & Jaeger, 2016, p. 34). We already know that gestures facilitate processing of new input, as indicated by the reduced N400s described above and in line with this definition. However, it is unknown whether this facilitation is the result of prediction (which starts before the speech is heard; i.e., prestimulus) rather than integration (which starts once the speech is heard; i.e., poststimulus).

This is because two aspects were missing from these previous studies (e.g., Osorio et al., 2024; Zhang et al., 2021). First, it is unclear whether the information in the gestures was visible before their corresponding speech in these earlier studies.^1^ If not, then the facilitation would not be the result of prediction but of concurrent multimodal semantic processing. Second, it is not known whether, if gestural information does precede related verbal information, this gestural information already impacts processing before the corresponding speech is heard (an essential aspect of the definition of prediction). A study using preceding gestures and measuring processing (i.e., behavior and neural activity) before the corresponding speech would provide crucial evidence to distinguish between prediction and integration and would help clarify the mechanisms underlying gestural facilitation.

## Current Study

The current study investigated whether comprehenders use speakers’ preceding gestures to predict upcoming meaning before it is heard in speech. In two experiments, participants listened to spoken questions asked by a virtual avatar. Virtual avatars allow for maximal control of designing multimodal utterances while also retaining good ecological validity (Peeters, 2019) and have been used to replicate various psycholinguistic effects, including linguistic prediction (e.g., Huizeling et al., 2022). Each question the avatar asked was accompanied by an iconic gesture (e.g., typing) or a control movement not conveying any semantic meaning (e.g., arm scratch) followed by a short silent pause and a target word (e.g., “type”). The gesture-target word combinations were taken from naturalistic, dyadic face-to-face conversations between acquaintances (ter Bekke et al., 2024b).

In a behavioral Cloze experiment, the questions were played up to the target word, and participants typed in how they expected the question to continue. We hypothesized that seeing the gestures would improve participants’ explicit predictions of upcoming target words not only compared with control movements but also a condition without any hand movements.

In an EEG experiment with separate participants, we analyzed prestimulus neural activity in the pause before the target word. Specifically, we looked at neural oscillations in the alpha (8–12 Hz) and beta (13–20 Hz) frequency ranges because reductions in their power have been found to be a marker of anticipation (e.g., Gastaldon et al., 2020; Prystauka & Lewis, 2019; Wang et al., 2018). The short silent pause was necessary for obtaining sufficient and clean oscillation data. If gestures are used for prediction, then gestures should reduce prestimulus alpha and beta power compared with control movements. Moreover, after the target word is heard, the N400 should be reduced for gestures versus control movements, indicating facilitated semantic processing (e.g., Kutas & Hillyard, 1984).

## Research Transparency Statement

### General disclosures

**Conflicts of interest:** All authors declare no conflicts of interest. **Funding:** This research was supported by European Research Council CoG Grant 773079 (to J. Holler) and the Max-Planck-Gesellschaft Minerva Fast Track Fellowship (to L. Drijvers). **Artificial intelligence:** No AI-assisted technologies were used in this research or the creation of this article. **Ethics:** This research was approved by the Radboud University Social Sciences Faculty Ethics Committee (approval code: ECSW-2018-135).

### Study disclosures

**Preregistration:** The research aims/hypotheses, methods and analysis plan of the EEG experiment were preregistered on July 12, 2022 on https://aspredicted.org/dwbq-95p8.pdf. The EEG data were collected from July 13, 2022, to October 28, 2022. There were no deviations from the preregistration. Neither the EEG preprocessing steps nor the Cloze experiment were preregistered. **Materials:** Because of an editorial error that did not make clear that on publication all study materials would need to be shared, the authors have been permitted to delay full public access to the video stimuli so they can be used in the authors’ other projects. The video stimuli are currently stored under embargo on OSF and will automatically become available on January 1, 2026, at https://osf.io/zjegm/files/osfstorage. In the interim period, the stimuli are available on request from the corresponding author for the purposes of inspection or replication only. Example stimuli (https://osf.io/25pvn/files/osfstorage) and the question set (https://osf.io/tuvq5) are publicly available. **Data:** All primary data are publicly available (https://osf.io/tuvq5): the Cloze data (https://osf.io/ykjgq/files/osfstorage), EEG data (https://osf.io/j58vu/files/osfstorage), and memory data (https://osf.io/j82ac/files/osfstorage). **Analysis scripts:** All analysis scripts are publicly available (https://osf.io/tuvq5): the Cloze scripts (https://osf.io/ykjgq/files/osfstorage), EEG scripts (https://osf.io/j58vu/files/osfstorage), and memory scripts (https://osf.io/j82ac/files/osfstorage). **Computational reproducibility:** The computational reproducibility of the results has been independently confirmed by the journal’s STAR team.

## Method

### Participants

For the Cloze experiment, 60 right-handed native Dutch speakers (53 women, 6 men, 1 other; *M*_age_ = 21 years, *SD* = 3) participated to reach 20 data points per item per condition,^2^ and 40 different right-handed native Dutch speakers (26 women, 14 men; *M*_age_ = 23 years, *SD* = 4) participated^3^ in the EEG experiment to reach 20 data points per item per condition. Participants had normal hearing and normal (or corrected-to-normal) vision; did not report any neurological, motoric, or language-related disorders; gave informed written consent before the experiment; and received financial compensation or course credits. The experiments were approved by the Radboud University Social Sciences Faculty Ethics Committee.

### Stimuli

Participants watched videos of a female virtual avatar asking questions. Each question contained a target word. The target words were originally produced during questions, together with a corresponding iconic gesture, in a Dutch corpus of natural face-to-face conversations (ter Bekke et al., 2024b). The avatar’s questions were designed such that the target word was sentence-final whenever possible and embedded in a question that could stand alone and did not require the original conversation context. Moreover, the target word was not highly predictable on the basis of the sentence context (to prevent ceiling effects), the gesture and target word were important for understanding the question, and it was possible for a speaker to briefly pause before the target word. The final question set (see the Supplemental Material available online) contained a mix of personal and factual questions that could mostly be answered with “yes” or “no.”

Each gesture-target word pair (*n* = 60; e.g., wiggling fingers depicting the act of typing plus the verb “type”) was used in two questions (e.g., “How old were you approximately when you learned to type?” and “Is it practical in this society to be good at typing?”), resulting in 120 questions. An actress, a native Dutch speaker speaking in a neutral accent, recorded the audio in a soundproof booth. She was instructed to ask the questions as in conversation in a slow tempo and with a pause before each target word. Speech before and after the pause was annotated in Praat (Boersma, 2001), cutting off clicks or voiceless breaths. The sound clips were then intensity-scaled to 70 dB and recombined with a pure silence of 920 ms (created in Praat) in between.

For each question, two videos were made: one with the avatar producing an iconic gesture and one with a control hand movement. For the gesture videos, the gestures from the corpus were recreated using Blender (Version 2.93.8). The gestures were adapted to always start and end with the hands in the same rest position in the lap. Sometimes the gestures were slightly adapted to better suit the questions, for example, regarding time (e.g., shorten a long hold), location, or direction. Crucially, the core meaning of the gesture always remained and continued to match the target word.

The control movements were six grooming movements (elbow scratch, jaw wipe, neck scratch, palm rub, forearm scratch, and hand scratch). They started and ended in the same rest position as the gestures. Moreover, they were matched with the gestures on important kinematic features (for details, see the Supplemental Material). Thus, any differences between the conditions in the EEG data were unlikely to be caused by kinematic differences. Note that this is also why we used a control condition with grooming movements rather than no hand movements: to isolate the processing of meaningful gestures in the EEG rather than mere movement perception.

**Fig. 1.**
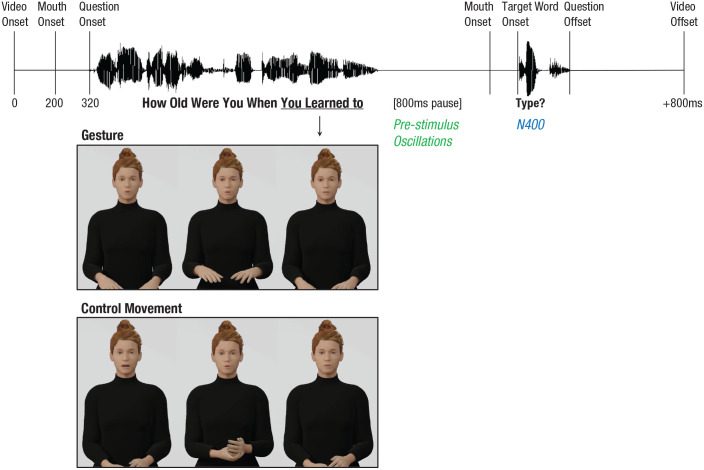
Schematic stimulus overview.

The avatar’s mouth movements were created on the basis of audio using Blender Rhubarb Lipsync. We created a custom mouth shape for the silent pause, with lips slightly open to indicate the avatar would continue speaking. Finally, each question was combined with the mouth and hand movements. All hand movements ended right before the silent pause. Mouth movements led audio by 120 ms, which is in line with the 100- to 300-ms lead found in natural speech (Chandrasekaran et al., 2009) and looked natural for our stimuli. Thus, the 920-ms silent pause consisted of 800 ms of silence without visible speech (our prestimulus analysis window) followed by 120 ms of silence with visible speech (Fig. 1). The prestimulus window was 800 ms (as in, e.g., Gastaldon et al., 2020; Terporten et al., 2019; Wang et al., 2018) to measure enough cycles of alpha oscillations. To avoid abrupt breaks, we added a static avatar for 200 ms before the question onset and 800 ms after the question offset. Twelve practice items were created in the same way. These items contained the same control movements as the experimental items (each one presented once) but different gestures (also from the corpus).

### Procedure

#### Cloze experiment

To investigate whether seeing the gestures improved participants’ explicit predictions of upcoming target words, 60 participants (who did not participate in the EEG experiment; for details, see Participants section) performed a Cloze experiment (online Gorilla experiment; Anwyl-Irvine et al., 2020). Participants were instructed that they would watch videos in which a virtual avatar would ask them questions. The video would suddenly stop even though the question had not yet been finished. It was the participants’ task to type in how they thought the question would continue. Participants could watch each video only once and were encouraged to type in their first intuition. The videos stopped after 50 ms of silent pause to avoid abrupt stops.

For this experiment, we also created a version of each video with no hand movements. This way we could check that seeing the control movements had the same impact on explicit predictions as seeing no hand movements. After 12 practice questions (four gesture, four control movement, four no hand movement), each participant saw 120 questions (40 per condition). Participants did not receive any feedback on their predictions. Overall, each question was seen by 20 participants in each condition.

#### EEG experiment

We explained to participants that they would watch videos containing a female virtual avatar who would ask them questions. They did not have to answer but only watch and listen attentively. Videos (25 frames per second) were presented using Presentation software (Version 23.0 10.27.21; Neurobehavioral Systems, Inc., Berkeley, CA) at a size of 1280 × 720 pixels and on a 1920 × 1080 monitor (120 Hz). Each trial started with a fixation cross (500 ms) that was followed by an empty gray screen (500–1,200 ms), the video (average: 5,400 ms), and another empty screen (1,000–1,500 ms). The next trial started automatically.

After 12 practice questions (six gesture, six control movement), the 120 experimental questions were presented in four blocks of 30 trials. The question order was pseudorandomized (practice and experimental), with the same condition occurring maximally twice in a row. Each question was seen in each block (1, 2, 3, 4) by one fourth of the participants, and in each condition (i.e., gesture, control movement) by half of the participants. For the two questions with the same target word (e.g., “typing”), each participant saw one with gesture and one with a control movement, with one block in between (i.e., Blocks 1 and 3 or Blocks 2 and 4). We counterbalanced the conditions in which each target word would be heard for the first time (50% gesture, 50% control movement), as well as the question context (50% Question 1, 50% Question 2).

After the main experiment, participants were immediately presented with an unexpected recognition memory test that served as an attention check. All participants scored above 50% (range: 73% to 100%), and therefore no participant was excluded. After the memory test, participants filled out several questionnaires. For details on the attention check and questionnaires, see the Supplemental Material.

### EEG recording

EEGs were continuously recorded from 32 silver/silver chloride electrodes, of which 31 were mounted in a 10-20 cap configuration, and one was placed on the right mastoid for offline re-referencing. The reference electrode was on the left mastoid (TP9), and the ground electrode was on the forehead (AFz). Electrode impedance was kept below 15 kOhm. Data were filtered online through a bandpass filter of 0.016 to 125 Hz and digitized with a sampling frequency of 500 Hz and resolution of 0.1 µV.

Triggers were sent at the start of target words, which typically started right after the silence. However, sometimes the target word (as taken from the corpus) consisted of multiple words (e.g., “very big backpack” for a gesture for which the backpack as well as its size were foregrounded in the movement). Then we time-locked the EEG to the word that most closely corresponded to the gesture (e.g., backpack). For seven items (of 120) this was not the first word after the silence.

### Data preprocessing and analysis

#### Cloze experiment

We analyzed participants’ predictions in terms of accuracy and, for the incorrect responses, semantic similarity (for preprocessing details, see the Supplemental Material). To calculate accuracy, we analyzed whether the exact target word was mentioned. To calculate semantic similarity between participants’ predictions and target words, we used word embeddings from subs2vec (van Paridon & Thompson, 2021). Because subs2vec can calculate only the similarity between two single words, we split each participant’s prediction for each item (e.g., prediction “wears a blouse” for the target word “shirt”) into separate words. For each prediction, the word with the highest semantic similarity to the target word was chosen, and its similarity score was used for analysis (e.g., “blouse”: 80% similarity to “shirt”).

We fitted (generalized) linear mixed-effects models for accuracy (correct, incorrect) and for semantic similarity (scores from 0 to 1). We used lme4 (Version 1.1-34; Bates et al., 2015) in R (Version 4.3.1; R Core Team, 2019). The condition was dummy-coded to compare gesture (0) to control movement (−1) and no hand movement (−1). The conditions control movement and no hand movement were compared post hoc using Tukey’s method in emmeans (Lenth, 2019). For details on random-effects structures and convergence issues, see the Supplemental Material.

#### EEG experiment

EEG data were preprocessed with FieldTrip (Version 20220626; Oostenveld et al., 2010) in MATLAB (Version R2020a; The MathWorks, Inc., Natick, MA). First, the data were segmented into epochs from −1.5 to 3.0 s relative to the target word onset. Next, the data were re-referenced to the average of the left and right mastoids. A notch filter removed noise components at 50, 100, and 150 Hz, and then the data were run through a bandpass filter of 0.1 to 100 Hz. We identified artifacts with a semiautomatic rejection routine. On average, we excluded 9.4% of the trials per participant (11 of 120). For one participant, an electrode was excluded and replaced with the average of its neighbors (as defined via a template). We applied independent component analysis to remove additional noise components associated with blinks or other eye movements. On average, 1.3 components were removed per participant (range: 0–4).

##### Prestimulus oscillations

To analyze prestimulus alpha and beta power modulations, time-frequency analysis was performed for the silent window (−920 to −120 ms before target word onset). Alpha (8–12 Hz) and beta (13–20 Hz) power were calculated for each trial per condition using a fast Fourier transform between 2 and 30 Hz and by applying a 500-ms Hanning window in frequency steps of 1 Hz and time steps of 10 ms. For each participant, each condition’s average power was baseline-corrected by subtracting the average power during a baseline window of +2,500 to +2,700 ms after the target word onset (using the “absolute” option in FieldTrip’s function ft_freqbaseline). During this baseline, participants were simply waiting for the next video to appear.

##### N400

To analyze N400 amplitude, data were run through an additional low-pass filter of 30 Hz and then baseline-corrected on the basis of a window of −300 to −100 ms before the target word onset. The average event-related potentials (time-locked to target word onset) were computed separately for the gesture and control movement conditions for each participant. We statistically analyzed the 300 to 700 ms after the target word onset.

##### Statistical analysis

We applied three nonparametric cluster-based permutation tests (Maris & Oostenveld, 2007) to compare the gesture and control movement conditions on prestimulus alpha power, prestimulus beta power, and N400 amplitude. First, a one-tailed dependent-samples *t* test was executed for every participant for every data point of the two conditions. For alpha power and N400 amplitude, data points were time by electrode. For beta power, data points were time by electrode by frequency.^4^ All adjacent data points that exceeded the preregistered threshold of 0.05 were grouped into clusters. For each cluster, the *t* statistics were summed to calculate the cluster-level statistics. A Monte Carlo permutation distribution was then created by randomly assigning a participant’s average to one of the conditions (5,000 times) and calculating the largest cluster-level statistic for every permutation. Cluster-level statistics were calculated for the actual data and compared against the distribution created by these 5,000 permutations. For alpha and beta power, clusters in the lowest 5% of the distribution were considered significant (i.e., one-tailed test for power decrease for gestures vs. control movements). For N400 amplitude, clusters in the highest 5% of the distribution were considered significant (i.e., one-tailed test for less negative N400 for gestures vs. control movements).

Finally, we also tested whether a trial’s prestimulus power predicted its poststimulus N400 amplitude. Such a pattern would be in line with prestimulus power reflecting prediction and poststimulus N400 amplitude reflecting prediction error. For details on the single-trial data preprocessing and analysis, as well as additional single-trial results, see the Supplemental Material.

## Results

### Gestures improved explicit predictions

**Fig. 2.**
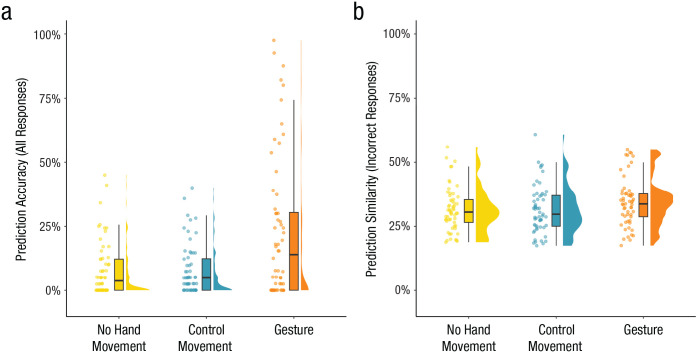
Gestures improved explicit predictions. Gestures improved (a) prediction accuracy and (b) semantic similarity of incorrect predictions compared with no hand movements and compared with control movements.

In the Cloze experiment, overall 12.9% (*SD* = 33.5) of the target words were predicted correctly (Fig. 2a). People more often predicted the correct target word when they had seen preceding gestures (23.0%) compared with control movements (7.8%; β = 0.80, *SE* = 0.31, *z* = 2.60, *p* < .01) and compared with no hand movements (7.8%; β = 0.97, *SE* = 0.30, *z* = −3.23, *p* < .01). There was no difference in prediction accuracy between the control movements and no hand movements (β = 0.17, *SE* = 0.21, *z* = −0.79, *p* = .709).

Even when participants did not predict the exact target word, the same pattern of results was found (Fig. 2b). Incorrect predictions were more similar to target words when participants had seen preceding gestures (34.0%) compared with control movements (31.2%; β = 0.03, *SE* = 0.01, *t*(56.32) = 3.76, *p* < 0.01) and compared with no hand movements (31.3%; β = 0.03, *SE* = 0.01, *t*(59.07) = 4.03, *p* < .01). There was no difference between the control movements and no hand movements (β = −0.00, *SE* = 0.00, *t* = −0.54, *p =* .851).

### Gestures reduced alpha and beta power before the target word

**Fig. 3.**
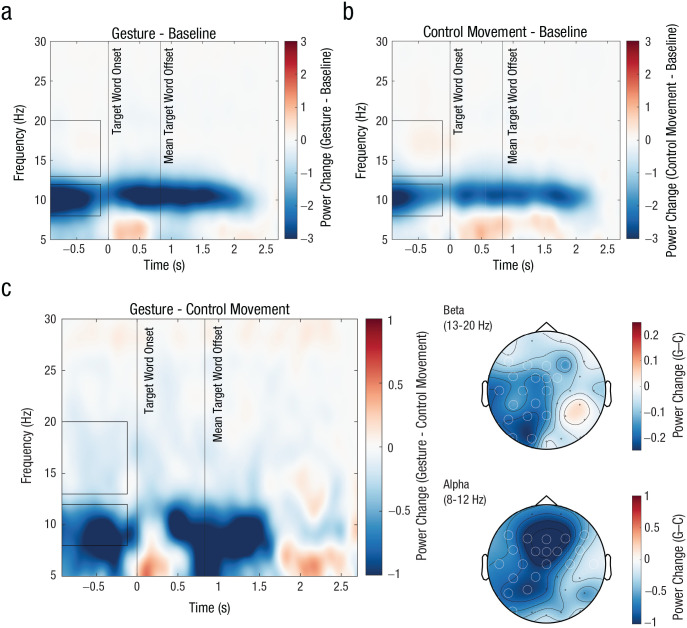
Time-frequency plots for the EEG experiment. The plots show the oscillatory power (a) in the gesture condition compared with baseline, (b) in the control movement condition compared with baseline, and (c) in the gesture condition compared with the control movement condition. In the beta frequency range between 13 and 20 Hz (top rectangles), there was a small power decrease for (c) gestures compared with control movements, which appeared to have been driven by a larger power increase (b) in the control movement condition. For a clearer visualization of the beta effects, see Figure SM1 in the Supplemental Material. In the alpha frequency range between 8 and 12 Hz (bottom rectangles), power decreased both (a) in the gesture and (b) control movement conditions but much more strongly (c) for the gesture condition. The (c, right) topographic distributions display the power changes (gesture − control movement) in the alpha and beta frequency ranges and the electrodes that were part of the significant clusters (white circles).

In the EEG experiment, gestures reduced alpha and beta oscillatory power during the silent pause before the target word. The cluster-based permutation test for the alpha frequency band revealed a significant alpha power suppression for gestures compared with control movements during the complete silent pause (from −920 to −120 ms; cluster *p* = .003; Fig. 3). Similarly, the cluster-based statistics for the beta frequency band revealed a significant beta power suppression for gestures compared with control movements during the complete silent pause (from −920 to −120 ms; cluster *p* = .009; Fig. 3).

### Gestures reduced the N400 at the target word

**Fig. 4.**
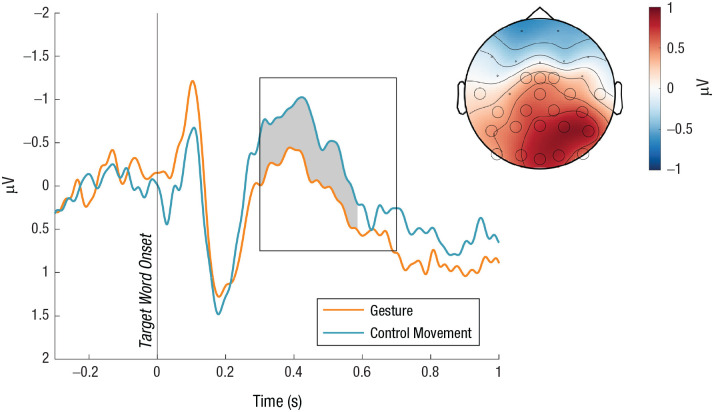
N400 event-related potentials averaged across electrodes that were part of the significant cluster. The rectangle indicates the time window of analysis (300–700 ms), and the gray shade indicates the time window that was part of the significant cluster (300–588 ms). The topographic distribution on the top right displays the difference between the conditions (gesture − control movement) and the electrodes that were part of the significant cluster (black circles).

When the target word was heard, gestures reduced the N400 event-related potential. The cluster-based permutation test revealed a less negative N400 for gestures compared with control movements from 300 to 588 ms after the target word onset (Fig. 4; cluster *p =* .015).

### Link between prestimulus power and N400 amplitude

On a single-trial level, neither prestimulus alpha power (β = 0.01, *SE* = 0.03, *t* = 0.34, *p =* .737) nor prestimulus beta power (β = −0.02, *SE* = 0.02, *t* = −0.81, *p =* .419) predicted the subsequent N400 amplitude.

## Discussion

Our study provides the first behavioral and neural evidence that comprehenders use speakers’ iconic gestures to predict upcoming meaning. In the Cloze experiment, gestures improved explicit predictions of upcoming target words. Moreover, the EEG experiment showed that gestures reduced prestimulus alpha and beta power, indicating anticipation (e.g., Prystauka & Lewis, 2019), and reduced N400 amplitudes, demonstrating facilitated semantic processing of target words (e.g., Kutas & Hillyard, 1984).

Our experiment allowed us to distinguish between gestures facilitating *prediction* (before the speech is heard) and *integration* (once the speech is heard) by using gestures that preceded their corresponding speech and by measuring neural activity before this speech was heard. Previous work did not investigate these two aspects for measuring prediction. For example, Zhang et al. (2021) showed that gestures reduced the N400 while participants listened to short narratives, which they concluded was “probably because they can support prediction” (p. 8). This interpretation matches our findings, and this study provides important evidence that gestures can modulate the surprisal of individual words. However, by measuring the N400 during the target word, prediction and integration mechanisms cannot be distinguished. The same holds for Osorio et al. (2024), who quantified the surprisal of each word in a multimodal narrative and measured whether the brain’s response to surprisal, an N400-like response, was modulated by gestures. Moreover, for both studies it is unclear whether the gestural information actually preceded the corresponding words. This also holds for an eye-tracking study by Silverman et al. (2010), who used gestures that did not precede speech enough for prediction to be possible. Finally, Skipper (2014) did use preceding gestures and found less activity in the auditory cortex after hearing words preceded by gestures, but again prestimulus processing was not measured. Our results thus provide the first evidence that preceding gestures already impact processing before the corresponding speech is heard, in line with prediction.

Predictive language processing is therefore multimodal in that both preceding speech and communicative gestures are used for prediction. Although some researchers have acknowledged the possibility of visual information being used as a cue for prediction (e.g., Huettig, 2015; Kuperberg & Jaeger, 2016; Pickering & Gambi, 2018), they referred only to visual scenes. Theories of linguistic prediction should take account of communicative bodily signals as an information source for prediction (Holler & Levinson, 2019; Skipper, 2014) to capture how language is used and processed in face-to-face interaction, the most natural environment of human language.

Gestures improving predictions of upcoming meaning is a possible mechanism via which gestures facilitate language processing, leading to faster comprehension and responding (ter Bekke et al., 2024a). This is crucial in face-to-face conversations, in which people rapidly take turns at talking. We do not claim that prediction is the only mechanism: Even when gestures do not precede their corresponding information in speech, they may still facilitate language processing by adding or emphasizing semantic information, for example. Future research is needed to gain a better understanding of the various mechanisms that may explain gestures’ facilitatory function.

Our N400 findings also have implications regarding speech-gesture integration. Some studies have suggested that gesture and speech need to occur close together for integration to occur (e.g., Habets et al., 2011; Obermeier & Gunter, 2015; Obermeier et al., 2011), whereas others have shown that integration still happens when the gesture is much earlier than the speech (e.g., 2 s before; Fritz et al., 2021). Our early gestures did not overlap even with their target words but were nevertheless integrated (indexed by the N400), in line with this wider window of speech-gesture integration. An important question for further research is whether there is an optimal time window for gestures, early enough to allow for the prediction of upcoming meaning but close enough for integration to occur as well, and how this time window relates to the gesture-speech timing found in natural conversations (e.g., ter Bekke et al., 2024b).

Moreover, our study provides the first evidence that comprehenders integrate speech and gestures from virtual avatars. This is essential knowledge regarding the changing nature of human communication and is driven by technological innovations and the increased use of avatars and human-like robots. To ensure that such artificial agents are readily understood, and in a human-like way, they should communicate not only with speech but also with meaningful hand gestures. The semantic integration of the virtual avatar’s speech and gestures also means that virtual avatars are a promising method for studying multimodal communication, allowing for good ecological validity (e.g., using gestures from conversations, increasingly photorealistic avatars) and excellent experimental control (e.g., precise timing, controlled kinematics; Peeters, 2019).

Specifically, future research could use avatars to investigate how other visual signals impact the prediction of various aspects of language (Holler & Levinson, 2019). At lower levels, comprehenders can use statistical regularities between preceding mouth movements and their corresponding sounds (Chandrasekaran et al., 2009) to predict these upcoming sounds (Arnal et al., 2011). Similarly, comprehenders might also use rhythmic gestures, eyebrow movements, or head nods to predict that a word or syllable will be emphasized prosodically. Importantly, visual signals might also be used to make higher level predictions about communicative intentions (Holler & Levinson, 2019; Trujillo & Holler, 2023), such as identifying utterances as questions or statements on the basis of early eyebrow movements (Nota et al., 2023) or even more detailed intention attributions on the basis of complex signals (Trujillo & Holler, 2024). Much more work is necessary to explore the overall hypothesis that comprehenders use multimodal signals to make predictions about how utterances will unfold (Holler & Levinson, 2019).

It is important to acknowledge a few limitations that warrant future exploration. First, in our stimuli the gestures preceded their target words more than they do in natural conversations (e.g., ter Bekke et al., 2024b), with silent pauses in between gestures and target words. Although this design was necessary to measure the relatively slow prestimulus oscillations in a controlled manner and does capture the natural gesture-before-speech timing that endows gestures with their predictive potential (ter Bekke et al., 2024b), future studies could use alternative methods to measure prediction on the basis of more precise naturalistic timing (e.g., visual-world eye-tracking paradigm with gestures; Silverman et al., 2010). Second, our data cannot tell us what types of representations comprehenders were predicting based on the gestures—that is, only conceptual meaning, or also specific words and the sounds associated with them? Third, we did not find that individual differences as measured in the current study affected gesture processing in our data (see the Supplemental Material). Future work could look into other dimensions of interindividual variation, such as working memory (e.g., Özer & Göksun, 2020), and investigate how these results may generalize to other participant groups. Finally, we found no relationship between a trial’s prestimulus alpha or beta power and its poststimulus N400 effect. We cannot conclude at this point whether this was because of insufficient statistical power or has implications for our functional interpretations of prestimulus alpha and beta power. Future research is necessary to establish whether and how prestimulus alpha and beta power are related to poststimulus N400 amplitude on a trial-by-trial basis during predictive language processing.

To conclude, we showed for the first time that comprehenders use speakers’ iconic hand gestures, which typically precede speech, to predict upcoming meaning. This means that the temporal organization of the multiple layers of semantic information available when hearing and seeing a speaker significantly alters the processes that underpin language comprehension. Our findings underline the importance of investigating human language in environments that capture core aspects of how language has evolved and is used most, namely in face-to-face interaction, in which language is multimodal.

## Supplemental Material

sj-docx-1-pss-10.1177_09567976251331041 – Supplemental material for Co-Speech Hand Gestures Are Used to Predict Upcoming Meaning Supplemental material, sj-docx-1-pss-10.1177_09567976251331041 for Co-Speech Hand Gestures Are Used to Predict Upcoming Meaning by Marlijn ter Bekke, Linda Drijvers and Judith Holler in Psychological Science

## Transparency

*Action Editor:* Vishnu Sreekumar

*Editor:* Simine Vazire

*Author Contributions*

**Marlijn ter Bekke:** Conceptualization; Formal analysis; Investigation; Methodology; Visualization; Writing – original draft.

**Linda Drijvers:** Conceptualization; Formal analysis; Methodology; Supervision; Writing – review & editing.

**Judith Holler:** Conceptualization; Formal analysis; Funding acquisition; Methodology; Supervision; Writing – review & editing.

**Artificial Intelligence:** No AI-assisted technologies were used in this research or the creation of this article.

**Ethics:** This research was approved by the Radboud University Social Sciences Faculty Ethics Committee (approval code: ECSW-2018-135).

**Open Practices:** Study disclosures. Preregistration: The research aims/hypotheses, methods and analysis plan of the EEG experiment were preregistered on July 12, 2022 on https://aspredicted.org/dwbq-95p8.pdf. The EEG data were collected from July 13, 2022 to October 28, 2022. There were no deviations from the preregistration. Neither the EEG preprocessing steps nor the Cloze experiment were preregistered. Materials: Because of an editorial error that did not make clear that on publication all study materials would need to be shared, the authors have been permitted to delay full public access to the video stimuli so they can be used in the authors’ other projects. The video stimuli are currently stored under embargo on OSF and will automatically become available on January 1, 2026 at https://osf.io/zjegm/files/osfstorage. In the interim period, the stimuli are available on request from the corresponding author for the purposes of inspection or replication only. Example stimuli (https://osf.io/25pvn/files/osfstorage) and the question set (https://osf.io/tuvq5) are publicly available. Data: All primary data are publicly available (https://osf.io/tuvq5): the Cloze data (https://osf.io/ykjgq/files/osfstorage), EEG data (https://osf.io/j58vu/files/osfstorage), and memory data (https://osf.io/j82ac/files/osfstorage). Analysis scripts: All analysis scripts are publicly available (https://osf.io/tuvq5): the Cloze scripts (https://osf.io/ykjgq/files/osfstorage), EEG scripts (https://osf.io/j58vu/files/osfstorage), and memory scripts (https://osf.io/j82ac/files/osfstorage). Computational reproducibility: The computational reproducibility of the results has been independently confirmed by the journal’s STAR team.

## Notes

1. For a notable exception, see Hintz et al. (2023).

2. This was based on previous work (Terporten et al., 2019) that used a similar EEG paradigm but with constraining speech in which 12 data points were used per item per condition. Because we expected the predictive effect of naturally occurring gestures to be smaller than that of sentence contexts designed to be highly constraining, we increased this number to 20. For consistency, we also used the same amount of data points per item per condition for the Cloze experiment.

3. One additional participant was tested but not included because they reported having a diagnosis of attention-deficit/hyperactivity disorder.

4. For alpha power, we averaged across the 8- to 12-Hz frequency range. For beta power, we did not average across the larger 13- to 20-Hz range to prevent hiding effects that maybe present only in part of this range (e.g., 16–20 Hz).
