# Supplementary material for: Co-Speech Hand Gestures Are Used to Predict Upcoming Meaning
Source: Psychol Sci. 2025 Apr 22;36(4):237–48. doi: 10.1177/09567976251331041 (PMC13043028; doi:10.1177/09567976251331041)
Supplement: sj-docx-1-pss-10.1177_09567976251331041 – Supplemental material for Co-Speech Hand Gestures Are Used to Predict Upcoming Meaning [file sj-docx-1-pss-10.1177_09567976251331041.docx]

Supplemental Material

**Title:** Co-speech hand gestures are used to predict upcoming meaning

**Authors:** Marlijn ter Bekke, Linda Drijvers, Judith Holler

# Supplemental methods

## Question details

For the questions and their English translations, please see the supplementary spreadsheet on <https://osf.io/tuvq5>.

## Movement kinematics

The control movements were six grooming movements that were matched with the gestures on several kinematic features. The movements were matched on handedness (gestures: 51.7% right-handed, 18.3% left-handed, 30.0% two-handed; control movements: 50% right-handed, 16.7% left-handed, 33.3% two-handed) and average duration (gestures: 1537 ms; control movements: 1566 ms). To match the movements on average peak velocity and average distance travelled by the hands, these values were calculated for each control movement and each gesture using OpenPose (Cao et al., 2021) and a script by Trujillo and colleagues (2019). The control movement animations were then tweaked, until ultimately the control movements involved comparable peak velocity (only 6.0% lower on average than the gestures) and distance travelled by the hands (only 7.4% higher on average).

## Memory test

After watching all the videos, participants in the EEG experiment were immediately presented with an unexpected recognition memory test that served as an attention check. Participants read 30 questions from the avatar where the target word had been changed, and 30 unchanged questions. They had to indicate whether they had heard this exact question or not via button press. Target word changes involved changes in meaning (i.e., no synonyms) that did not result in odd questions. Note that as the questions were presented in written form, no gestures were seen during the memory test. All participants scored above 50% (range: 73-100%) and therefore, following our pre-registration, no participant was excluded.

## Questionnaires

After the memory test, participants in the EEG experiment filled out the Empathy Quotient (EQ; Baron-Cohen & Wheelwright, 2004), Autism-Spectrum Quotient (AQ; Baron-Cohen et al., 2001; Hoekstra et al., 2008), and the Actions and Feelings Questionnaire (AFQ; van der Meer et al., 2022; Williams et al., 2016). Participants scored typically on the questionnaires (EQ: *M =* 44.5 vs. reference *M* = 42.1 (*SD =* 10.6); AQ: *M* = 18.0 vs. reference *M* = 17.6 (*SD* = 7.0); AFQ: *M* = 35.1 vs. reference *M* = 32.1 (*SD* = 4.9)). Participants’ scores did not predict how much they were affected by gesture in the EEG experiment.

## Cloze data preprocessing

Before calculating accuracy and semantic similarity, we preprocessed participants’ predictions by putting compound words together when they should be (e.g., “neer gestoken” becomes “neergestoken”), replacing abbrevations with their full words (e.g., “TV” becomes “televisie”), replacing English words with their translations (e.g., “research” becomes “onderzoek”), and fixing spelling mistakes (e.g., “baggage” becomes “bagage”).

### Semantic similarity preprocessing

After splitting each prediction (e.g., “wears a blouse”) into separate words, we removed articles, “and” and “or”. Moreover, target words consisting of multiple words were either replaced with their key meaning (*n* = 7 target words out of 60; e.g., “long hair” became “hair”), or excluded from this specific analysis when choosing one word was impossible (*n* = 3 target words out of 60; e.g., “heen en weer” – back and forth). Furthermore, target words unrecognized by *subs2vec* were replaced with their best alternative (e.g., a synonym or less specific term, *n* = 4 target words out of 60). Unrecognized words in the participants’ predictions were excluded, as it was unfeasible to reliably replace all these instances (*n* = 126 words out of 11798). In the resulting dataset, for each prediction the word with the highest semantic similarity to the target word was chosen and its similarity score was used for analysis.

## Linear mixed effects model convergence issues

We first used maximal random effects structures. In case of singular fits, we simplified the models in a step-by-step approach, each time removing random effects explaining close to 0 variance or with correlations close to +/- 1. None of the simplified models resulted in different conclusions from the maximal models. However, if the simplified model had a significantly poorer fit to the data, as established using likelihood ratio tests (alpha = 0.2, following Matuschek et al., 2017), we used the more complex model instead and accepted the singularity warning. This occurred only twice (Cloze experiment accuracy and semantic similarity analyses).

# Supplemental analyses

## Memory test

As pre-registered, we explored whether participants remembered the target words better if they had been preceded by a gesture compared to a control movement. We fitted linear-mixed effects models for accuracy (correct, incorrect) and reaction times (in milliseconds, log-transformed) using the lme4 package (version 1.1.34; Bates et al., 2015) in R (version 4.3.1; R Core Team, 2019). Condition was sum-to-zero contrast-coded (Gesture 1, Control movement -1).

For 87.1% of the trials in the memory test (*SD* = 33.5%), participants correctly recognized whether a target word had changed or not (Gesture: *M* = 88.0%, *SD* = 32.5%; Control movement: 86.3%, *SD* = 34.5%). Comparing the conditions, there was no difference in accuracy (*β* = -0.08, *SE* = 0.10, *z* = -0.88, *p* = 0.382), nor in reaction times (*β* = -0.01, *SE* = 0.01, *t* = -0.91, *p* = 0.367).

## Beta frequency visualization


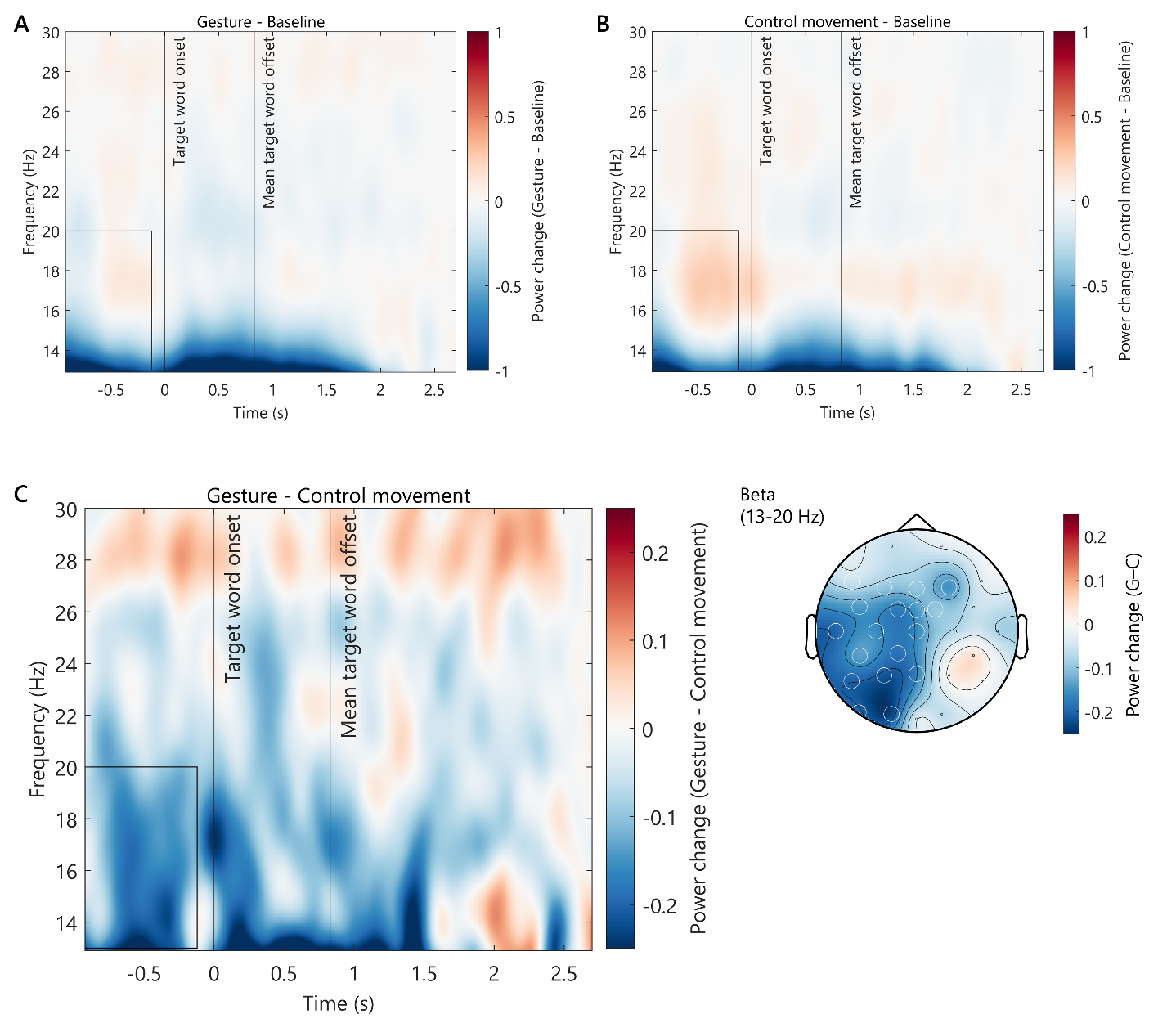


*Figure SM1*. Time-frequency plots showing the oscillatory power in the Gesture condition compared to baseline (panel A), in the Control movement condition compared to baseline (panel B) and in the Gesture condition compared to the Control movement condition (panel C). To better visualize the effects in the beta frequency range (rectangles, 13-20 Hz), the frequencies are shown from 13-30 Hz. For visualization of the effects in the alpha frequency range, please refer to Figure 3 in the main article. There is a small beta power decrease for gestures compared to control movements (panel C), which appears to be driven by a larger power increase in the Control movement condition (panel B). The right side of panel C shows the topography of the power changes (Gesture – Control movement) in the beta frequency range, with the electrodes that were part of the significant clusters marked with white circles.

## Single-trial data preprocessing and analysis

As pre-registered, we also performed additional regression analyses based on single-trial data. First, we tested whether gestures especially modulated pre-stimulus power and N400 amplitude if they improved explicit predictions more in the Cloze experiment. We expected that if certain gestures improved explicit predictions more (e.g., typing gesture vs. a circular tracing gesture), they may also modulate pre-stimulus power and N400 amplitude more. Second, we tested whether gestures especially modulated pre-stimulus power and N400 amplitude if the target word was less predictable based on speech only. This analysis tested whether gestures are processed differently depending on the predictability of the speech. Third, we also tested whether a trial’s pre-stimulus power predicted its post-stimulus N400 amplitude. The results of this analysis are reported in the main manuscript, but the information below on single-trial data preprocessing and analysis also applies to it.

Based on the significant clusters from the cluster-based permutation tests, we extracted for each trial a single value for alpha power, beta power and N400 amplitude. For the oscillatory power, we calculated the power spectra again as for the main analyses, only without averaging across trials (i.e., keeptrials = “yes” in Fieldtrip’s function ft_freqanalysis). Next, for alpha power we averaged across the pre-registered frequency range of 8-12 Hz and then averaged across the time-electrode data points that were part of the significant cluster. For beta power, we averaged across the time-electrode-frequency data points that were part of the significant cluster. For N400 amplitude, we averaged the amplitude values across the pre-registered window of 300-700 ms after target word onset. We then averaged across the electrodes that were part of the significant cluster.

We fitted linear-mixed effects models using the lme4 package (version 1.1.34; Bates et al., 2015) in R (version 4.3.1; R Core Team, 2019). Condition was sum-to-zero contrast-coded (Gesture 1, Control movement -1). The single-trial N400 amplitude, alpha power and beta power values were z-standardised. See Supplemental methods for details on random effects structures and convergence issues.

### Relation between gesture predictiveness and EEG

To test whether gestures especially modulated pre-stimulus power and the N400 if they improved explicit predictions more in the Cloze experiment, we used models with the interaction between Condition (Gesture, Control movement) and Gesture predictiveness (continuous) as predictor. For each item, Gesture predictiveness was calculated as the average Cloze score in the Gesture condition minus the average Cloze score in the Control movement condition. Because it was possible that the Gesture predictiveness score coincidentally correlated with another variable unrelated to gesture (e.g., question duration, with gestures improving predictions more in longer questions), the Control movement condition was added in an interaction. A significant interaction between Gesture predictiveness and Condition would reflect the effect of the Gesture predictiveness truly due to seeing the gestures.

The models revealed that gesture predictiveness did not impact the effect of condition on pre-stimulus alpha power (*β* = -0.01, *SE* = 0.01, *t* = -0.54, *p* = 0.589), nor on pre-stimulus beta power (*β* = -0.02, *SE* = 0.01, *t* = -1.24, *p* = 0.215), nor on N400 amplitude (*β* = 0.02, *SE* = 0.01, *t* = 1.16, *p* = 0.246). Exploratory analyses showed that gesture predictiveness based on semantic similarity also did not influence the effect of condition on pre-stimulus alpha power (*β* = -0.01, *SE* = 0.01, *t* = -0.50, *p* = 0.621), nor on pre-stimulus beta power (*β* = -0.02, *SE* = 0.01, *t* = -1.14, *p* = 0.256), nor on N400 amplitude (*β* = 0.02, *SE* = 0.02, *t* = 1.09, *p* = 0.278).

### Relation between target word predictability and EEG

To test whether gestures especially modulated pre-stimulus power and the N400 compared to control movements when the target word was less predictable based on speech only, we used models with the interaction between Condition (Gesture, Control movement) and Target word predictability (continuous) as predictor. For each item, Target word predictability was calculated as the average Cloze score in the No hand movement condition.

The models revealed that target word predictability did not impact the effect of condition on pre-stimulus alpha power (*β* = -0.01, *SE* = 0.01, *t* = -0.43, *p* = 0.668), nor on pre-stimulus beta power (*β* = -0.01, *SE* = 0.01, *t* = -0.38, *p* = 0.702), nor on N400 amplitude (*β* = 0.02, *SE* = 0.2, *t* = 1.23, *p* = 0.222). Exploratory analyses showed that target word predictability based on semantic similarity also did not influence the effect of condition on pre-stimulus alpha power (*β* = -0.00, *SE* = 0.01, *t* = -0.30, *p* = 0.766), nor on pre-stimulus beta power (*β* = -0.01, *SE* = 0.01, *t* = -0.65, *p* = 0.519), nor on N400 amplitude (*β* = 0.01, *SE* = 0.02, *t* = 0.83, *p* = 0.410).

# References

Baron-Cohen, S., & Wheelwright, S. (2004). The empathy quotient: An investigation of adults with asperger syndrome or high functioning autism, and normal sex differences. *Journal of Autism and Developmental Disorders*, *34*(2), 163–175. https://doi.org/10.1023/B:JADD.0000022607.19833.00

Baron-Cohen, S., Wheelwright, S., Skinner, R., Martin, J., & Clubley, E. (2001). The autism-spectrum quotient (AQ): Evidence from Asperger syndrome/high-functioning autism, males and females, scientists and mathematicians. *Journal of Autism & Developmental Disorders*, *31*(1), 5–17. https://doi.org/10.1023/A:1005653411471

Cao, Z., Hidalgo, G., Simon, T., Wei, S.-E., & Sheikh, Y. (2021). OpenPose: Realtime multi-person 2D pose estimation using part affinity fields. *IEEE Transactions on Pattern Analysis and Machine Intelligence*, *43*(1), 172–186. https://doi.org/10.1109/TPAMI.2019.2929257

Hoekstra, R. A., Bartels, M., Cath, D. C., & Boomsma, D. I. (2008). Factor structure, reliability and criterion validity of the Autism-Spectrum Quotient (AQ): A study in Dutch population and patient groups. *Journal of Autism and Developmental Disorders*, *38*(8), 1555–1566. https://doi.org/10.1007/s10803-008-0538-x

Matuschek, H., Kliegl, R., Vasishth, S., Baayen, H., & Bates, D. (2017). Balancing Type I error and power in linear mixed models. *Journal of Memory and Language*, *94*, 305–315. https://doi.org/10.1016/j.jml.2017.01.001

Trujillo, J. P., Vaitonyte, J., Simanova, I., & Özyürek, A. (2019). Toward the markerless and automatic analysis of kinematic features: A toolkit for gesture and movement research. *Behavior Research Methods*, *51*(2), 769–777. https://doi.org/10.3758/s13428-018-1086-8

van der Meer, H. A., Sheftel-Simanova, I., Kan, C. C., & Trujillo, J. P. (2022). Translation, cross-cultural adaptation, and validation of a Dutch version of the actions and feelings questionnaire in autistic and neurotypical adults. *Journal of Autism and Developmental Disorders*, *52*(4), 1771–1777. https://doi.org/10.1007/s10803-021-05082-w

Williams, J. H. G., Cameron, I. M., Ross, E., Braadbaart, L., & Waiter, G. D. (2016). Perceiving and expressing feelings through actions in relation to individual differences in empathic traits: The Action and Feelings Questionnaire (AFQ). *Cognitive, Affective, & Behavioral Neuroscience*, *16*(2), 248–260. https://doi.org/10.3758/s13415-015-0386-z
